# Changes in patient experience associated with growth and collaboration in general practice: observational study using data from the UK GP Patient Survey

**DOI:** 10.3399/bjgp20X713429

**Published:** 2020-11-03

**Authors:** Lindsay JL Forbes, Hannah Forbes, Matt Sutton, Katherine Checkland, Stephen Peckham

**Affiliations:** Centre for Health Services Studies, University of Kent, Canterbury.; Division of Population Health, Health Services Research and Primary Care, University of Manchester, Manchester.; Division of Population Health, Health Services Research and Primary Care, University of Manchester, Manchester.; Division of Population Health, Health Services Research and Primary Care, University of Manchester, Manchester.; Centre for Health Services Studies, University of Kent, Canterbury.

**Keywords:** family practice, general practice, observational study, organisational models, policy

## Abstract

**Background:**

For the last few years, English general practices — which are, traditionally, small — have been encouraged to serve larger populations of registered patients by merging or collaborating with each other. Meanwhile, patient surveys have suggested that continuity of care and access to care are worsening.

**Aim:**

To explore whether increasing the size of the practice population and working collaboratively are linked to changes in continuity of care or access to care.

**Design and setting:**

This observational study in English general practice used data on patient experience, practice size, and collaborative working. Data were drawn from the English GP Patient Survey, NHS Digital, and from a previous study.

**Method:**

The main outcome measures were the proportions of patients at practice level reporting positive experiences of both access and relationship continuity of care in the GP Patient Survey. Changes in proportions between 2013 and 2018 among practices that had grown and those that had, roughly, stayed the same size were compared, as were patients’ experiences, categorised by whether or not practices were working in close collaborations in 2018.

**Results:**

Practices that had grown in population size had a greater fall in continuity of care (by 6.6%, 95% confidence interval = 4.3% to 8.9%), than practices that had roughly stayed the same size, after controlling for other factors. Differences in falls in access to care were smaller (4.3% difference for being able to get through easily on the telephone; 1.5% for being able to get an appointment; 0.9% in satisfaction with opening hours), but were statistically significant. Practices collaborating closely with others had marginally worse continuity of care than those not working in collaboration, and no differences in access.

**Conclusion:**

Larger general practice size in England may be associated with slightly poorer continuity of care and may not improve patient access. Close collaborative working did not have any demonstrable effect on patient experience.

## INTRODUCTION

Traditionally, general medical practices in England are relatively small: in 2018, most practices were led by a partnership of GPs (mean: 3–4 full-time equivalents [FTEs]), employing a multidisciplinary team and delivering primary care to a mean of 8000 registered patients.^1^ Since 2014, the idea that general practices should work at scale — in other words, work together to deliver services to larger populations — has been a central element of English health-service policy. Working at scale was intended to enable practices to innovate, make savings, better support staff, become more resilient, have a stronger voice in negotiations, and facilitate longer opening times.^2^ The evidence to suggest that serving a larger population of patients achieves these aims is, however, limited, as is the evidence about possible negative consequences.^3^^–^^5^

Between 2014 and 2018, guidance from NHS England on how practices should work together was not prescriptive. It set out a number of options, including merging with other practices to form new, single organisations or forming groups linked by different types of agreement, in which individual practices retained variable degrees of autonomy.^6^^,^^7^ By 2018, the average number of patients registered per practice was growing and mergers joining several practices into single business units were becoming more common.^8^^–^^10^ Practices were also increasingly participating in collaborative groups,^11^ although there is evidence that some had been working in this way for several years before 2014.^12^^,^^13^ Although there have been some surveys and evaluations of these organisational models,^3^^,^^12^^–^^14^ NHS England has not collected data systematically on the extent of the implementation.^15^ However, from previous work by the authors of this article and that of others, it was clear that a small proportion of these collaborative groups were working very closely together, with a common strategy and shared risk.^13^^,^^15^

In early 2019, NHS guidance set out plans that were more specific on how general practices should work together, announcing that primary care networks, serving 30 000–50 000 patients in total, should be formed.^16^^,^^17^ These were intended to be made of up of between six and eight practices, with each covering an identifiable geographical footprint. The constituent practices were intended to remain as autonomous organisations; they were not expected to merge legally, but were expected to work together to deliver specific functions collaboratively.^18^ The formation of primary care networks in the summer of 2019 made the need for evidence of the effects of increasing the size of practice populations to whom primary care is delivered more urgent.

A key concern about increasing the size of primary care organisations is loss of continuity of care, which is highly valued by patients^9^^,^^10^ and one of the core values of good primary care.^19^^,^^20^ Continuity of care encompasses the relationship between practitioner and patient (relationship continuity), coordination and teamwork (management continuity), and availability of records (information continuity).^21^ Better relationship continuity is associated with lower mortality,^22^ fewer hospital admissions,^23^ fewer emergency department attendances,^24^ and better patient experience;^19^^,^^25^ however, relationship continuity of care has been falling over recent years in England.^26^

**Table:** How this fits in

Primary medical care in England has, traditionally, been delivered by small general practices. Over the last few years, and particularly since 2014, practices have been encouraged to grow or to work collaboratively to serve larger populations. This study found no convincing evidence that practices that have grown have better access to care but their relationship continuity of care may be worse. Practices that were collaborating closely in 2018 did not have better or worse access or continuity of care than those that were not.

Close collaborative working and merging may threaten relationship continuity because patients may be more likely to see an unfamiliar health professional. If care is delivered across several sites, information continuity may become a challenge if records are not freely shared, which is particularly likely if practices are not legally joined into one organisation.^27^ Relationship continuity may be more challenging if there is limited information continuity.

This study aimed to explore whether the fall in continuity of care over the last few years^26^ could be related to the policy encouragement to increase practice size and examine the effect on access to care.

At the time of writing, data on the effect of the formation of primary care networks on access or continuity were available. As such, the authors focused on two types of organisational changes — large growth in registered populations and models in which practices were working together in strong collaborative groups.

## METHOD

Two sets of analyses were carried out to examine the effect of increases in the size of a general practice population, either through practice growth or working in close collaboration, on access and continuity of care. The first analysis — of the effect of practice growth — examined what happened to access and continuity of care in practices that had grown in registered population between 2013 and 2018 compared with those that had stayed, approximately, the same size over that period. The second analysis — of the effect of collaboration — examined whether there was any difference in access and continuity of care in practices that were working in close collaborative groups but not merging formally, compared with those that were not. This second analysis was necessarily cross-sectional using 2018 data as there were no data available about working in strong collaborative groups in 2013.

### Identifying growing practices

For the first analysis, the difference in size of the registered population for each practice with >1000 patients between April 2013 and March 2018 was calculated using NHS Digital datasets.^28^^,^^29^ The change in registered population ranged from a fall of 5620 patients to an increase of 60 392 patients; the mean was an increase of 840 patients per practice. For the analysis to be meaningful, it was necessary to identify practices for which the increase in practice size would represent a significant change in ways of working. There is no universally accepted way of defining such a meaningful increase in practice size but the authors considered that an increase of >2000 patients (roughly equivalent to gaining one additional FTE GP) would be meaningful for a small practice, but less so for a very large practice. Practices were, therefore, categorised as having had a meaningful increase in size if their registered population had increased by >2000 patients and by >20% (the percentage increase was arbitrary but determined a priori). As such, to be classified as having had a meaningful increase in size, a practice with 4000 patients at baseline would have to have increased its population to 6001 patients, and a practice with 20 000 patients at baseline would have to have increased their population to 24 001 patients. The comparison group was made up of those practices where the size of the registered population in 2018 was around the same as in 2013 (defined as having a population size in 2018 of ≥80% and ≤120% of the population size in 2013).

The small number of practices for which >30% of the registered population was aged 15–24 years in 2013 or 2018 were excluded, as these were all based in universities. They all had a very large registered population and most had grown in size by >20%; university practices work at this scale because they have a large number of healthy, young patients who are not resident for all of the year and, in addition, the practices may have grown because of the rising student population^30^ and not because of the benefits envisaged by national policy. It was, therefore, considered that they were not relevant to this analysis. Practices with <3000 patients at baseline were also excluded; this was because they were likely to represent partnerships of fewer than two FTE GPs and were, therefore, atypical.

### Identifying practices working in collaboration

The authors used a dataset they had developed about the extent to which practices had been working in close collaboration in early 2018, as set out in their previous article.^15^ They found that it was possible to identify three different kinds of practice, namely:

- those working closely in collaboration to deliver core GP services (as defined in national contracts with NHS England extant at the time^31^^,^^32^) collectively to >30 000 patients. Some of these groups were very large, single-business entities, and others were working as part of ‘super-partnerships’ or multisite organisations, with separate identities but shared a strategy and shared risk;^13^
- those working loosely in collaboration to deliver services over and above core GP services (for example, extended opening times, specialist clinics delivered in general practice) collectively to >30 000 patients. Practices in this group retained more autonomy and did not share strategy or risk; and
- those not working in collaboration.

### Access and continuity-of-care variables

To assess patients’ perspectives of access and continuity of care, responses to the GP Patient Survey^33^ were used; this is the only source of routine data on this issue that is available for all English practices. Funded by NHS England, the GP Patient Survey is a national standardised postal questionnaire survey of a very large sample of adults (∼800 000 in 2017)^34^ who have been registered with a practice for at least 6 months. It has been running at least once a year for >10 years and uses stratified random sampling such that patients at every practice are selected.^35^ The survey asks about a range of issues including overall satisfaction, how easy it was to get an appointment, opening hours, waiting times, experience of seeing a preferred GP, experience of the last appointment, current state of health, and management plans.

Five questions were selected a priori for this analysis. Of those, only one question in the survey reflected patient experience of continuity of care: *‘How often do you see or speak to the GP you prefer?’* This question was applicable to responders who replied *‘yes’* to the question: *‘Is there a GP you usually prefer to see or speak to?’* (46% of patients in 2017).^34^ The response categories were: *‘always or almost always’*; *‘a lot of the time’*; *‘some of the time’*; *‘never or almost never’*; and *‘not tried at this GP surgery’.* The outcome variable used was the proportion of patients in each practice with a positive response — namely, *‘always or almost always’* or *‘a lot of the time’.*

Access to care was measured using three questions, the possible responses for which are given in parentheses:

- *‘How satisfied are you with the hours that your GP surgery is open?’ (‘very satisfied’, ‘fairly satisfied’, ‘neither satisfied nor dissatisfied’, ‘fairly dissatisfied’, ‘very dissatisfied’).* The outcome variable was the proportion for each practice answering ‘very satisfied’ or ‘fairly satisfied’.
- *‘Generally, how easy is it to get through to someone at your GP surgery on the phone?’ (‘very easy’, ‘fairly easy’, ‘not very easy’, ‘not at all easy’, ‘haven’t tried’).* The outcome variable was the proportion for each practice answering *‘very easy’* or *‘fairly easy’*.
- *‘Were you able to get an appointment to see or speak to someone, the last time you wanted to see or speak to a GP or nurse at your GP surgery?’ (‘Yes’, ‘yes but I had to call back closer to or on the day I wanted the appointment’, ‘no’, ‘can’t remember’).* The outcome variable was the proportion for each practice answering *‘yes’* or *‘yes but I had to call back closer to or on the day I wanted the appointment’.*

The authors also examined responses to the question about overall experience: *‘Overall, how would you describe your experience of your GP surgery?’ (‘Very good’, ‘fairly good’, ‘neither good nor poor’, ‘fairly poor’, ‘very poor’).* The outcome variable was the proportion for each practice answering *‘very good’* or *‘fairly good’.*

Data from the 2013 and 2017 waves of the GP Patient Survey were used (in both surveys, data were collected between January and March)^36^ to reflect the time period over which working at scale became a central part of English policy. Data from 2018 were not used as the questions changed in that year, making trends more difficult to interpret. The percentages of people giving positive responses had been weighted by the research organisation (Ipsos MORI) to allow for unequal probabilities of selection, non-response, and non-representativeness.^35^

### Statistical analysis

Analyses were conducted using Stata (version 14). For both sets of analyses, the assumptions of linear regression models were sufficiently met. The relationships between continuous covariates and the dependent variables appeared roughly linear, the variances were similar in the comparison groups, and the residuals followed near-normal distributions.

#### Effect of practice growth

The authors examined the differences between the practices that had grown by >20% and the comparison group in terms of:

- age of registered population — percentage aged <5 years, percentage aged ≥75 years;
- sex — percentage male;
- rurality — rural or urban;
- region — North of England (North), the Midlands, London and the South of England (London and South);
- level of socioeconomic deprivation — mean Index of Multiple Deprivation (IMD) score for the practice, based on patients’ home postcodes; the scores were estimated using data from the 2011 Census relating to the Lower Layer Super Output Area (LSOA) of residence of registered patients by practice^37^ and the IMD calculated in 2015 for LSOA^38^); and
- prevalence of long-term conditions — percentage with longstanding illness based on responses to the GP Patient Survey.

To examine the association between change in practice size and change in access, continuity, and overall experience, five linear regression analyses were carried out. The outcome variables were mean changes between 2013 to 2017 in mean percentages with positive responses to the five GP Patient Survey questions. These were, for all five questions, approximately normally distributed. Included in the model were the variables associated with change in practice size; that is, percentage aged <5 years, percentage aged ≥75 years, region, rurality, level of socioeconomic deprivation, and percentage with long-term conditions. These have been found by other researchers to be related to performance on the GP Patient Survey.^39^^–^^43^ The values at different time points on the covariates were very highly correlated; the most recent data available were used in the models.

#### Effect of collaboration

Differences between the three groups of practices (close collaboration, loose collaboration, and no collaboration) in terms of age of registered population, rurality, region, socioeconomic status, and prevalence of long-term conditions have been reported previously.^15^

The outcome variables were the percentages reporting positive responses to the five 2017 GP Patient Survey questions. These were approximately normally distributed and were compared between practices working, and not working, in close collaboration using linear regression; the same covariates were used as for the previous set of analyses. To examine the effect of previous GP Patient Survey responses on the associations, the equivalent percentage of positive responses from 2013 were included as covariates in the models.

## RESULTS

The number of practices in England with list sizes of >1000 patients was 7971 in April 2013^28^ and 7162 in March 2018.^1^ Eight university practices were excluded. Both sets of analyses were carried out on the 7089 practices with data available on NHS Digital about practice size for 2013 and 2018, as well as data for both the 2013 and 2018 GP Patient Surveys.

### Distribution of GP Patient Survey responses

In the whole sample of 7089 practices, the mean percentage of patients reporting being able to see their preferred GP fell from 63.0% to 56.0% over the 5-year period. Good overall experience fell slightly from 87.0% to 85.0%. Finding it easy to get through to the practice on the telephone fell from 77.0% to 71.0%, being satisfied with opening hours fell from 80.0 to 77.0%, and being able to make an appointment fell only slightly from 86.0% to 84.0%.

**Figure 1.**
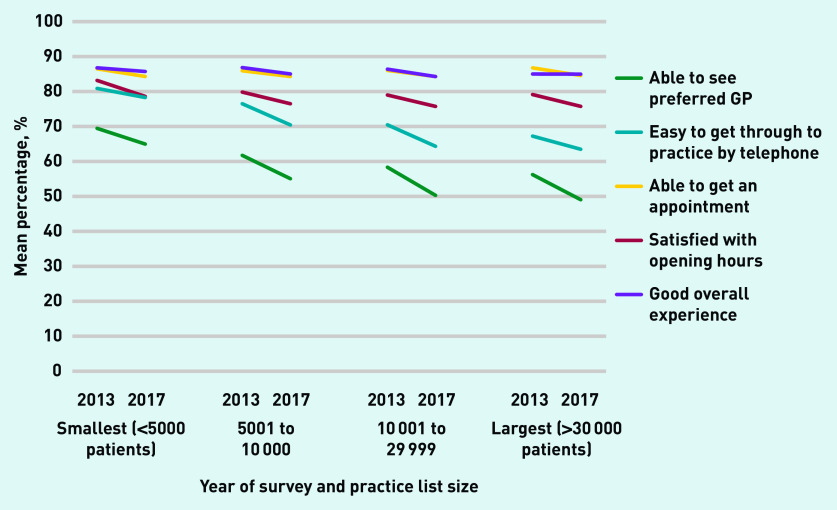
**Mean percentages of patients giving positive responses to GP Patient Survey questions in 2013 and 2017, by size of registered population in March 2018.**

Figure 1 shows the change in mean percentages of positive responses to the GP Patient Survey over the period by size of registered practice population in 2018. Good overall experience, being able to get an appointment, and satisfaction with opening hours were high for all sizes of practice, with small falls over the time period. Being able to get through on the telephone and being able to see a preferred GP appeared to deteriorate over time, with the lowest percentages applying to the largest practices.

### Effect of practice growth

Between 2013 and 2018, the registered population size had grown (by >2000 patients and >20%) in 644 practices and had stayed approximately the same (within 80% and 120% inclusive of the population in 2013, as previously defined) in 5106 practices (from a total of 7089 practices). The remaining 1339 practices had either fallen in size by >20% *n* = 55), had a 2013 population of ≤3000 patients (*n* = 924), or had grown by ≤2000 patients (*n* = 788); the numbers do not total 1339 because many practices fell into more than one category.

The characteristics of the practices that had grown between 2013 and 2018, and those that stayed about the same size, are shown in Table 1. Proportionally, practices that had grown had fewer older people, more young children, fewer patients with longstanding illnesses, and were more socioeconomically deprived than practices that stayed about the same size, but no differences were large (although they were statistically significant). Practices that had grown were, however, much less likely to be rural and much more likely to be in London and the South of England than practices that had stayed about the same size.

**Table 1.** Characteristics of practices by practice growth between 2013 and 2018^a^

**Characteristic**	**Practices with no growth^b^ (*n* = 5106)**	**Practices with growth^c^ (*n* = 644)**	***P-*value for difference**
Mean list size, *n*			
2013	8322	8722	
2018	8627	14 087	

Mean increase in list size 2013–2018, %	3.8	69.6	

Aged ≥75 years, mean %			
2013	7.9	6.3	<0.001
2018	8.3	6.3	<0.001

Aged <5 years, mean %			
2013	6.0	6.5	<0.001
2018	5.5	6.0	<0.001

Male, mean %			
2013	49.7	49.7	0.30
2018	49.9	50.1	0.89

Longstanding illness, mean %			
2013	53.8	51.5	<0.001
2018	54.1	51.1	<0.001

Index of Multiple Deprivation 2015 score	22.2	24.6	<0.001

Rural, %	16.8	7.3	<0.001

Region, %			
London and South	39.1	53.9	
Midlands	30.5	24.8	
North	30.5	21.3	<0.001

a *For practices with >3000 patients in 2013,* n *= 5750, excluding practices with fall in patient numbers of >20%.*

b Practices whose registered population in 2018 was +/−20% inclusive of that of 2013.

c Practices whose registered population increased by >20% and >2000 patients between 2013 and 2018.

Practices that had grown had larger falls in percentage change in positive GP Practice Survey responses than practices that had stayed approximately the same size (Table 2). Although the differences were small, they were all statistically significant and were only marginally changed by adjusting for the possible confounding variables. The only difference that was >5% when adjusted, however, was for being able to see a preferred GP.

**Table 2.** Positive responses to GP Patient Survey questions, by practice growth for practices^a^

**Positive response**	**Practices with no growth,%^b^ (*n* = 5106)**	**Practices with growth, %^c^ (*n* = 644)**	**Adjusted difference in % change between 2013 and 2017^d^ (95% CI)**
Able to see preferred GP^d^			
2013	62.5	59.3	
2017	55.6	48.9	
Change between 2013 and 2017^e^	−9.2	−14.2	−6.6 (−8.9 to −4.3)

Easy to get through to practice on telephone			
2013	75.7	77.2	
2017	69.7	69.1	
Change between 2013 and 2017^e^	−7.3	−12.2	−4.3 (−5.9 to −2.6)

Able to get an appointment			
2013	86.2	85.5	
2017	84.6	82.6	
Change between 2013 and 2017^e^	−1.6	−3.1	−1.5 (−2.2 to −0.8)

Satisfied with opening hours			
2013	79.7	81.0	
2017	76.3	76.7	
Change between 2013 and 2017^e^	−3.9	−4.7	−0.9 (−1.8 to −0.1)

Good overall experience			
2013	86.9	86.8	
2017	85.2	83.1	
Change between 2013 and 2017^e^	−1.5	−3.9	−2.2 (−3.0 to −1.4)

a *For practices with >3000 patients in 2013, excluding practices where the number of patients had fallen by >20%,* n *= 5750.*

b Practices whose registered population in 2018 was +/−20% inclusive of that of 2013.

c Practices whose registered population increased by >20% and >2000 patients between 2013 and 2018.

d Controlling for percentage aged ≥75 years in 2018, percentage aged <5 years in 2018, percentage with longstanding illness in 2017, mean Index of Multiple Deprivation 2015 score, region (North, Midlands, London and South), urban/rural setting.

e Percentage change is calculated as percentage point change from 2013–2017 divided by the percentage in 2013.

### Effect of working in collaboration

Data were available on working in collaboration in early 2018 and from the 2017 GP Patient Survey for 6673 (94.1%) of 7089 practices: 206 were working in close collaboration, 3666 in loose collaboration, and 2801 were not working in collaboration.

In practices that were working in close collaboration, patients were 11.9 percentage points less likely to report being able to see their preferred GP than patients in practices not working in collaboration (45.1% versus 57.0%); however, this difference reduced to <4 percentage points after controlling for covariates, with the equivalent percentage of positive responses in 2013 having a particularly strong effect (Table 3). The differences for access and overall experience were only approximately one percentage point worse in practices working in collaboration compared with those that were not after adjusting for covariates (Table 3). This suggests that differences in patient experience between practices working in close collaboration and not working in collaboration were probably due to differences in population characteristics.

**Table 3.** Positive responses to GP Patient Survey questions, by closeness of collaborative working

**Positive response**	**No collaboration (*n* = 2801), %**	**Loose collaboration (*n* = 3666), %**	**Close collaboration (*n* = 206), %**	**Adjusted difference between close-collaboration and no-collaboration practices, % (95% CI)**	
				**Model 1^a^**	**Model 2^b^**
Able to see preferred GP	57.0	56.3	45.1	−8.7 (−11.3 to −6.1)	−3.6 (−5.6 to −1.6)
Easy to get through to practice on telephone	71.6	71.5	64.8	−3.1 (−5.6 to −0.6)	−0.9 (−2.7 to 0.8)
Able to get an appointment	85.0	84.3	81.2	−0.5 (−1.5 to 0.5)	−0.5 (−1.5 to 0.5)
Satisfied with opening hours	77.0	77.0	75.5	−0.5 (−1.8 to 0.8)	−0.6 (−1.7 to 0.5)
Good overall experience	85.6	85.3	81.0	−1.9 (−3.2 to −0.6)	−1.3 (−2.4 to −0.2)

a Model 1 includes percentage aged ≥75 years in 2018, percentage aged <5 years in 2018, percentage with longstanding illness in 2017, mean Index of Multiple Deprivation 2015 score, region (North, Midlands, London and South), urban/rural setting.

b Model 2 includes all Model 1 variables plus the equivalent percentage from the GP Patient Survey 2013.

*Totals practices,* n *= 6673.*

## DISCUSSION

### Summary

This study suggests that practices that had grown in size between 2013 and 2018 had no more improvement or deterioration in access or overall experience to an important degree than practices whose list size had remained approximately the same. Practices that had grown had a greater fall in patients’ reports of being able to see their preferred GP than those that had not, by 4.3% to 8.9%. Patients in practices that were working closely in collaboration in 2018 had worse measures of patient overall experience, access, and continuity than those that were not working in this way, but this was largely explained by differences in demographics, geography, and historical patterns of patient experience. The authors found some evidence that increasing practice size might have a negative effect on continuity of care, but no important effect on access to care. The analysis of the effect of working in close collaboration did not suggest any effects on patient experience.

### Strengths and limitations

To the authors’ knowledge, this is the first study that has attempted to assess how change in practice size and working in collaboration affect access and continuity of care. Other studies have examined quantitatively the effects of practice size on access and continuity of care in cross-sectional designs but not the effects of change in practice size.^4^

The only study of collaboration and continuity of care was a qualitative interview study asking for the perspectives of healthcare staff.^3^ All the datasets used had very few missing data. For the analysis of the effects of collaboration, the authors are confident that they identified a group of practices working closely in collaboration, but there may have been some misclassification of practices not working in collaboration.^15^

Whether the association between worse continuity of care and practice growth is causal is unclear. Practices may seek to grow because they are poorly performing in some way or have poor GP Patient Survey performance, and any positive effects of practice growth on patient experience could be slow to be realised. It is possible that the differences found may be due to residual confounding by unknown factors that are associated with both practice growth and changes in responses to the GP Patient Survey.

Access to care was measured using responses to three questions from the GP Patient Survey that examined three different elements of access; namely, getting through to the practice on the telephone, getting an appointment, and being satisfied with the practice’s opening hours. However, access to primary care is a complex construct and not easily defined or measured;^44^ it is possible that these questions do not comprehensively measure the experience of access for patients.

Continuity of care was measured using a single question from the GP Patient Survey, which aims to measure relationship continuity of care with a single GP. Although this has been associated with positive effects on mortality, quality of life, and hospital admissions,^22^^,^^23^^,^^25^^,^^45^ it reflects only perceptions of continuity with a single GP. It may be that satisfactory continuity of care that maximises benefits for individual patients may be achieved by providing team-based care that either includes other GPs or other health professionals. Moreover, relationship continuity of care is only part of the concept of continuity; it may be that seeing several different health professionals affords the same benefits as seeing a single GP if information continuity — that is, record sharing and handover — is good. Other measures of continuity of care that encompass a broader view have been developed,^46^^,^^47^ but these are likely to be too long to be incorporated into a routine survey of hundreds of thousands of people, without compromising the response rate. It is also possible that some patients are willing to sacrifice continuity of care in order to achieve better access; some groups of patients value continuity of care more than others.^48^

### Comparison with existing literature

An analysis of the effect of an initiative undertaken between 2008 and 2015, which aimed to fund practices to extend their opening hours, found that there were small positive effects on access and overall experience; however, the researchers did not report effects on continuity of care.^49^

A recent systematic review examining the implications of large, general practice collaborations in England found no quantitative studies of their effects on patient experience and evidence from just one qualitative case study suggesting detrimental effects on continuity of care.^3^

In line with the findings presented here, a systematic review in 2013 found that smaller practices achieved better patient-reported access to services and better continuity of care than larger practices. The studies included in the systematic review were cross-sectional, but found an inverse association between practice size and patient-reported access and continuity,^4^ and another cross-sectional 2014 study by the Institute for Fiscal Studies found that larger GP practices had lower patient experience scores (although not specifically continuity of care when compared with smaller practices).^5^

Larger general practice size in England may be associated with slightly poorer continuity of care and may not improve patient access; this supports the opinions of experts, who have warned that continuity of care will be a likely casualty of increasing access to general practice and growing practice size.^27^

### Implications for practice

North American studies suggest that small practices may deliver better patient experience more cheaply and have fewer hospital admissions.^50^^–^^53^ It is too early to evaluate the impact of initiatives implemented only in the last few years, and other influences — for example, part-time primary care staff, changes in skill mix in general practice, lower public sector spending — may have also had an impact on patient experience, but the study presented here provides no support for the view that larger practice size or close collaborative working improves access to care.

Negative effects on continuity of care are important, not only because of the high value placed on this element of general practice by patients,^48^ but also because of the evidence that it is associated with better health outcomes.^22^^,^^23^^,^^48^ Continuity of care is particularly important in the care of people with long-term conditions,^54^ who are a key priority in English health policy.^55^

The authors recommend better, more complete, timely, and meaningful routine data collection about models of collaborative working and practice growth; it may be that availability of data improves with the formation of primary care networks. Although effects on health, costs, safety, workforce, and evidence-based care are meaningful, it is also important to monitor the effects of organisational change in terms of the patient experience.

The current study also suggests that new ways of monitoring continuity of care for routine data collection should be considered, recognising that the concept encompasses more than the face-to-face relationship between two individuals; that is, one doctor and the patient.

It is hoped that primary care networks prioritise the recommendation of the *GP Partnership Review* and that they *‘operate in a way that … enables partners … to support continuity of high-quality, personalised holistic care’.*^56^
